# Longitudinal–Torsional Frequency Coupling Design of Novel Ultrasonic Horns for Giant Magnetostrictive Transducers

**DOI:** 10.3390/s24186027

**Published:** 2024-09-18

**Authors:** Khurram Hameed Mughal, Bijan Shirinzadeh, Muhammad Asif Mahmood Qureshi, Muhammad Mubashir Munir, Muhammad Shoaib Ur Rehman

**Affiliations:** 1 Robotics and Mechatronics Research Laboratory (RMRL), Department of Mechanical and Aerospace Engineering, Monash University, Melbourne, VIC 3800, Australia; khurram.mughal@monash.edu; 2 Mechanical Engineering Department, University of Engineering and Technology, Lahore 54890, Pakistan; asifqureshi1971@uet.edu.pk (M.A.M.Q.); mubashirmunir321@gmail.com (M.M.M.);; 3 Mechanical Engineering Department, The University of Lahore, Lahore 54000, Pakistan

**Keywords:** longitudinal–torsional coupled (LTC) vibrations, ultrasonic LTC horns, finite element analysis, robotic ultrasonic machining, amplitude ratio, giant magnetostrictive transducer

## Abstract

The use of advanced brittle composites in engineering systems has necessitated robotic rotary ultrasonic machining to attain high precision with minimal machining defects such as delamination, burrs, and cracks. Longitudinal–torsional coupled (LTC) vibrations are created by introducing helical slots to a horn’s profile to enhance the quality of ultrasonic machining. In this investigative research, modified ultrasonic horns were designed for a giant magnetostrictive transducer by generating helical slots in catenoidal and cubic polynomial profiles to attain a high amplitude ratio ($T_{A}/L_{A}$) and low stress concentrations. Novel ultrasonic horns with a giant magnetostrictive transducer were modelled to compute impedances and harmonic excitation responses. A structural dynamic analysis was conducted to investigate the effect of the location, width, depth and angle of helical slots on the Eigenfrequencies, torsional vibration amplitude, longitudinal vibration amplitude, stresses and amplitude ratio in novel LTC ultrasonic horns for different materials using the finite element method (FEM) based on the block Lanczos and full-solution methods. The newly designed horns achieved a higher amplitude ratio and lower stresses in comparison to the Bezier and industrial stepped LTC horns with the same length, end diameters and operating conditions. The novel cubic polynomial LTC ultrasonic horn was found superior to its catenoidal counterpart as a result of an 8.45% higher amplitude ratio. However, the catenoidal LTC ultrasonic horn exhibited 1.87% lower stress levels. The position of the helical slots was found to have the most significant influence on the vibration characteristics of LTC ultrasonic horns followed by the width, depth and angle. This high amplitude ratio will contribute to the improved vibration characteristics that will help realize good surface morphology when machining advanced materials.

## 1. Introduction

In recent years, numerous researchers have dedicated significant effort to the advancement of new materials characterized by a high strength-to-weight ratio. Examples include engineered ceramics, aluminum honeycomb and SiC/Al composites. These materials hold considerable utilization potential in high-performance engineering applications due to their lightweight nature and high strength [1,2,3]. However, high-quality machining of these materials poses a significant challenge to achieving the required precision for their widespread industrial application. Traditional machining methods often lead to undesirable outcomes such as excessive tool wear and the formation of burrs, chips and cracks in these advanced materials. Consequently, to fully leverage the potential of these materials, it becomes imperative to explore alternative non-traditional machining processes. One such method is rotary ultrasonic machining (RUSM), an advanced hybrid process that combines the elements of conventional machining with ultrasonic vibrations. By facilitating the intermittent contact between the work-piece and cutting tool, RUSM offers a promising approach for the efficient machining of advanced brittle composites, thereby enhancing their usability in various industrial applications.

**Figure 1:**
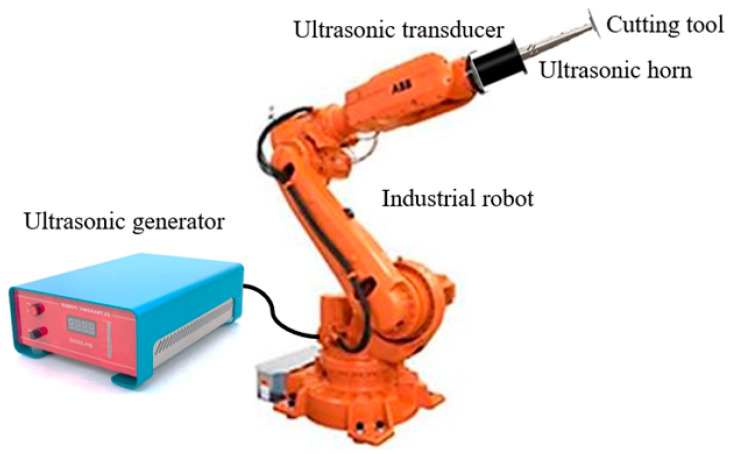
Generic representation of robotic ultrasonic cutting tool.

Various researchers have investigated the superiority of RUSM over conventional ultrasonic machining (USM) processes for advanced materials [4,5]. Thoe et al. emphasized the higher performance of RUSM compared to USM and observed the attainment of a higher material removal rate (MRR) [6]. Liu et al. used ultrasonic vibration-assisted microelectrochemical milling of micrometal parts to enhance machining quality, efficiency and precision [7]. Mughal et al. investigated the superiority of RUSM over conventional machining to improve the surface quality of Nomex honeycomb composite structures using various cutting tools [8,9]. Integrating an ultrasonic cutting system with an industrial robot provides more flexibility in accurate profiling, path planning and program generation as compared to conventional machine tools. The fundamental setup of robotic RUSM is illustrated in Figure 1. An ultrasonic generator converts a low-frequency electrical signal into high-frequency electrical output which is then transformed into mechanical vibrations by the ultrasonic transducer. However, the vibration amplitude at the transducer’s output end is insufficient for achieving effective machining characteristics. An ultrasonic horn is employed at the transducer end to amplify the vibration amplitude, ensuring high-quality machining of the work-piece material. A cutter is affixed to the horn’s output end to carry out the required cutting operation. The speed controller regulates the angular velocity of the cutting tool, whereas the working frequency is controlled by the ultrasonic generator. The ultrasonic vibrations of the cutting tool result in intermittent contact between the work-piece and cutter, leading to significantly enhanced machining quality with reduced cutting force [1,3]. The ultrasonic vibrations have been used effectively by many researchers in other engineering systems as well [10,11,12,13].

Significant attention has been paid by researchers to optimizing ultrasonic horn design as well as giant magnetostrictive transducer (GMT) design to attain good acoustic performance. Wang et al. analyzed a third-order Bezier horn to attain good vibration amplification, employing a multi-objective optimization algorithm and FEM [14]. Mughal et al. introduced a novel ultrasonic horn, evaluating its performance in terms of axial stiffness, modal frequencies, vibration amplification and several stresses against various horn profiles using ANSYS modal and harmonic analyses modules [15]. Li et al. designed a GMT using FEA [16]. Zhou et al. presented a generic amplitude prediction model for a GMT [17]. Wei et al. modelled a GMT using FEA [18]. Yang et al. optimized a GMT using a honeycomb panel for weight reduction [19].

Researchers have also identified the potential of longitudinal–torsional coupled (LTC) vibrations in enhancing the surface morphology of advanced materials in comparison to longitudinal vibrations. LTC vibrations are usually produced by introducing helical slots to the horn’s profile. Lin et al. [20] studied the characteristics of an LTC exponential horn, while Wang et al. [21] examined an LTC stepped horn with spiral grooves. Chen et al. studied the correlation between the resonant frequency and diagonal slits to examine LTC transformation efficiency using FEA [22]. Al-Budairi et al. evaluated LTC exponential horns for ultrasonic application [23]. Shahid et al. analyzed an LTC stepped horn with slanting grooves for ultrasonic application using an FEA coupled artificial neural network (ANN) technique [24]. Pang et al. studied an LTC stepped horn for RUSM [25]. Li et al. employed helical slots in the conical profile to develop an LTC horn with a large tool head [26]. Munir et al. analyzed novel LTC cubic and quadratic Bezier ultrasonic horns for the machining of hard, brittle materials using FEA [27].

Numerous studies have investigated the design of ultrasonic horns to achieve higher vibration amplification while minimizing stress levels. Researchers have explored ultrasonic horn designs of various geometries, including conical, stepped, exponential, Gaussian, catenoidal, quadratic Bezier, cubic Bezier and hybrid profiles, aiming to enhance vibration amplitude at the tool end for good machining quality and MRR. Helical slots have been incorporated into Bezier, exponential, conical and stepped horns to induce longitudinally and torsionally coupled vibrations, thereby improving RUSM characteristics. Stepped horns are prone to premature failure due to high stress concentrations at the transition section, while the optimal design of Bezier horns requires complex and time-consuming optimization procedures. Cubic polynomial ultrasonic horns provide good vibration amplification with low stress; however, their performance with helical slots to realize longitudinal–torsional coupled vibrations for robotic RUSM with a giant magnetostrictive transducer remains unexplored by researchers. This paper aims to address this gap by presenting the design and optimization of catenoidal and cubic polynomial horns with helical slots for a giant magnetostrictive transducer. The authors used the advantages of the cubic polynomial horn and generated helical slots to realize longitudinally and torsionally coupled vibrations. To achieve this objective, FEA was conducted to determine Eigenfrequencies, impedances, torsional and longitudinal amplitudes, various stresses and the amplitude ratio ($T_{A}/L_{A}$). The significant effect of several geometric factors on LTC vibration characteristics was also investigated, yet another unique aspect of this research work. Numerous amplitude ratios were obtained through FEA for each design, and optimal values with low stress levels were chosen and proposed to improve robotic RUSM with a giant magnetostrictive transducer. The numerical results were critically evaluated and compared with the literature for validation.

## 2. Materials and Methods

### 2.1. Analytical Model of LTC Ultrasonic Horn for Giant Magnetostrictive Transducer

Developing an analytical solution to the wave equation in realistic magnetostrictive ultrasonic transducers is challenging due to the involvement of different materials and the complex geometries of the transducer components, along with couplings and discontinuities. However, an analytical model can be developed using the equivalent circuit approach, which provides an efficient method for designing ultrasonic transducers [18]. The mechanical impedance of these parts is depicted by inductive ($Z_{L}$) and capacitive ($Z_{C}$) electrical impedances. These impedances can be calculated based on excitation frequency, geometrical dimensions and material properties from (1) and (2).

(1) $$Z_{M}=j\rho cA\tan\left(Sl_{p}/2\right)$$

(2) $$Z_{C}=-j\rho cA/\sin\left(Sl_{p}\right)$$

where ρ, c, A, S and $l_{p}$ are the density, speed of sound, area, wave number and wave path length, respectively.

**Figure 2:**
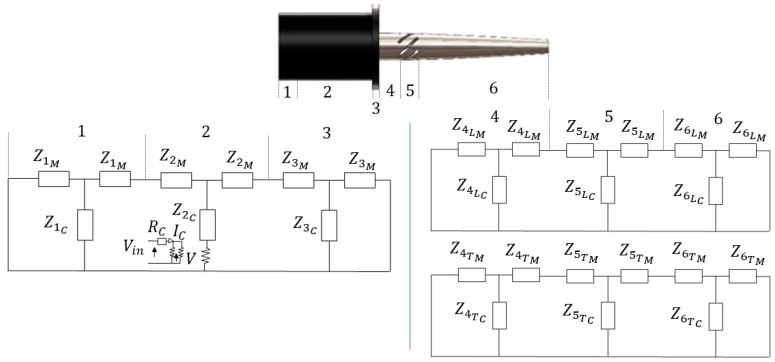
Equivalent circuit for LTC horn integrated with giant magnetostrictive transducer.

To apply these principles, the magnetostrictive transducer and LTC horn were divided into three regions each at points where there is a change in cross-sectional area, material or both, as shown in Figure 2. Equations (1) and (2) were then utilized to calculate the mechanical impedances for each region considering the assumption of constant strain, resulting in the equivalent circuit elements being added in series. Regions 1 and 3 are the rear and front portions of the transducer, whereas region 2 includes the magnetostrictive material. Regions 4 and 6 are the rear and front portions of the LTC ultrasonic horn, whereas region 5 includes the helical slots to produce longitudinal–torsional coupled vibrations. Although Equations (1) and (2) were applied for longitudinal vibrations, they can also be adapted for torsional vibrations. According to equivalent circuits, the mechanical impedances of a giant magnetostrictive transducer ($Z_{G_{m}}$) and an LTC horn for both longitudinal ($Z_{L_{m}}$) and torsional ($Z_{T_{m}}$) modes can be calculates as follows:

(3) $$Z_{G_{m}}=\left[\left(Z_{a_{m}}\times Z_{b_{m}}\right)/\left(Z_{a_{m}}+Z_{b_{m}}\right)\right]+Z_{2_{C}}$$

(4) $$Z_{L_{m}}=\left[\left(Z_{c_{m}}\times Z_{d_{m}}\right)/\left(Z_{c_{m}}+Z_{d_{m}}\right)\right]+Z_{5_{C}}$$

(5) $$Z_{T_{m}}=\left[\left(Z_{e_{m}}\times Z_{f_{m}}\right)/\left(Z_{e_{m}}+Z_{f_{m}}\right)\right]+Z_{5_{C}}$$

In Equations (3)–(5),

$$Z_{a_{m}}=\frac{Z_{1_{M}}\times Z_{1_{C}}}{Z_{1_{M}}+Z_{1_{C}}}+Z_{1_{M}}+Z_{2_{M}}\;,\;Z_{b_{m}}=\frac{Z_{3_{M}}\times Z_{3_{C}}}{Z_{3_{M}}+Z_{3_{C}}}+Z_{3_{M}}+Z_{2_{M}}$$

$$Z_{c_{m}}=\frac{Z_{4_{L_{M}}}\times Z_{4_{L_{C}}}}{Z_{4_{L_{M}}}+Z_{4_{L_{C}}}}+Z_{4_{L_{M}}}+Z_{5_{L_{M}}}\;,\;Z_{d_{m}}=\frac{Z_{6_{L_{M}}}\times Z_{6_{L_{C}}}}{Z_{6_{L_{M}}}+Z_{6_{L_{C}}}}+Z_{6_{L_{M}}}+Z_{5_{L_{M}}}$$

$$Z_{e_{m}}=\frac{Z_{4_{T_{M}}}\times Z_{4_{T_{C}}}}{Z_{4_{T_{M}}}+Z_{4_{T_{C}}}}+Z_{4_{L_{M}}}+Z_{5_{L_{M}}}\;,\;Z_{d_{m}}=\frac{Z_{6_{T_{M}}}\times Z_{6_{T_{C}}}}{Z_{6_{T_{M}}}+Z_{6_{T_{C}}}}+Z_{6_{T_{M}}}+Z_{5_{T_{M}}}$$

**Figure 3:**
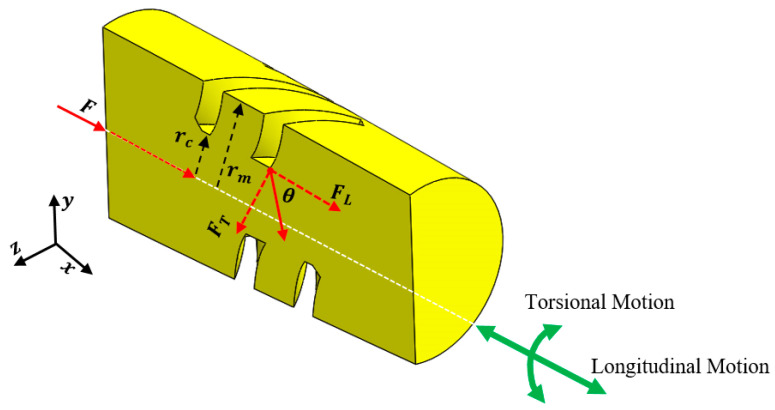
Section view of the novel LTC ultrasonic horn with longitudinal and torsional vibrations.

For an LTC ultrasonic horn as illustrated in Figure 3, (6) can be utilized to approximate the horn’s longitudinal amplitude $L_{A}\left(x,t\right)$ [15,27].

(6) $$\frac{\partial^{2}L_{A}\left(x,t\right)}{\partial x^{2}}+\frac{\partial^{2}L_{A}\left(x,t\right)}{\partial x^{2}}\frac{\partial }{\partial x}\ln A\left(x\right)=\frac{1}{{C_{L}}^{2}}\frac{\partial_{2}L_{A}(x,t)}{\partial t^{2}}$$

where A(x) is the horn’s cross-sectional area at any particular distance x and $c_{L}$ is the speed at which longitudinal vibrations propagate through the horn, which can be computed from $c_{L}=\sqrt{E/\rho }$ in terms of Young’s modulus (E).

For simple harmonic motion and wave number $S=\omega /c_{L}$, (1) becomes

(7) $$\frac{\partial^{2}L_{A}\left(x,t\right)}{\partial x^{2}}+\frac{\partial^{2}L_{A}\left(x,t\right)}{\partial x^{2}}\frac{\partial }{\partial x}\ln A\left(x\right)=S^{2}L_{A}\left(x,t\right)$$

Equation (8) gives the longitudinal amplitude of the ultrasonic horn by solving (7), while the horn’s torsional amplitude is estimated from (9) [20,27].

(8) $$L_{A}\left(x,t\right)=\left(k_{1}\sin Sx+k_{1}\cos Sx\right)/\sqrt{A\left(x\right)}$$

(9) $$\frac{\partial^{2}T_{A}\left(x,t\right)}{\partial x^{2}}+\frac{\partial^{2}T_{A}\left(x,t\right)}{\partial x^{2}}\frac{\partial }{\partial x}\ln I\left(x,r\right)=\frac{1}{c_{T^{2}}}\frac{\partial^{2}T_{A}\left(x,t\right)}{\partial t^{2}}$$

Here, I(x,r) and $T_{A}\left(x,t\right)$ are the polar moment of inertia and torsional amplitude of the ultrasonic LTC horn, respectively, and $c_{T}$ is the speed at which torsional vibrations propagate through the horn, which can be computed from $c_{T}=\sqrt{G/\rho }$ in terms of shear modulus (G). For simple harmonic motion and circular wave number $R=\omega /c_{T}$, (7) becomes

(10) $$\frac{\partial^{2}T_{A}\left(x,t\right)}{\partial x^{2}}+\frac{\partial^{2}T_{A}\left(x,t\right)}{\partial x^{2}}\frac{\partial }{\partial x}\ln I\left(x,r\right)=R^{2}T_{A}\left(x,t\right)$$

Equation (11) gives the torsional amplitude of the ultrasonic horn by solving (10).

(11) $$T_{A}\left(x,t\right)=\left(k_{1}\sin Sx+k_{1}\cos Sx\right)/\sqrt{I\left(r,x\right)}$$

Various stresses can be determined by using the following governing equations [27]:

(12) $$\partial \sigma_{r}/\partial r+\partial \sigma_{z}/\partial z+\left(\sigma_{r}-\sigma_{\theta }\right)/r+\rho \omega^{2}r=\rho \partial^{2}v_{r}/\partial t^{2}$$

(13) $$\partial \sigma_{r}/\partial r+\partial \sigma_{z}/\partial z+\tau_{rz}/r=\rho \partial^{2}v_{z}/\partial t^{2}$$

Here, $\sigma_{\theta }$, $\sigma_{r}$ and $\sigma_{z}$ are stresses in the LTC horn in the tangential (θ), radial (r) and longitudinal (z) directions, respectively. $\tau_{rz}$ represents in-plane shear stress, while $\rho \partial^{2}v_{r}/\partial t^{2}$ and $\rho \partial^{2}v_{z}/\partial t^{2}$ are radial and axial inertial forces, respectively.

The LTC vibrations are produced by introducing helical slots in the ultrasonic horn [23,24,25,26,27]. A constant inertial force F is applied by the giant magnetostrictive transducer on the LTC horn at regions without helical slots, which is converted into tangential ($F_{T}$) and longitudinal ($F_{L}$) components at the slots (Figure 3), as given by (14). $F_{L}$ initiates the longitudinal vibrations, whereas the moment M produced by $F_{T}$ is responsible for the torsional vibrations. $F_{T}$ and moment M are determined from (15) in terms of infinitesimal tangential force f for unit area dA at radial distance r, respectively [23,27].

(14) $$F_{L}=F\cos\theta ,\;\;\;\;\;\;\;\;\;F_{T}=F\sin\theta$$

(15) $$F_{T}=\int fdA,\;\;\;\;\;\;\;\;\;\;M=\int rfdA$$

The cross-sectional area for the combined slotted and solid core region (Figure 3) is

(16) $$\{\begin{array}{c} A_{1}=\pi r_{1}^{2}\;\;\;\;\;\;\;\mathrm{for}\;\;\;\;\;\;\;0<r_{1}\leq r_{c}\\ A_{2}=\pi r_{2}^{2}-\pi {\left(r_{2}-r_{c}\right)}^{2}=2\pi r_{c}r_{2}-\pi r_{c}^{2}\;\;\;\;\;\;\;\mathrm{for}\;\;\;\;\;\;\;r_{c}<r_{1}\leq r_{m} \end{array}$$

Differentiating (16) yields

(17) $$dA_{1}=2\pi r_{1}dr_{1},\;\;\;\;\;\;\;\;\;dA_{2}=2\pi r_{c}dr_{2}$$

Using (17) in (15), the following relation may be written as

(18) $$M=\overset{r_{c}}{\underset{0}{\int }}r_{1}f_{1}dA_{1}+\overset{r_{m}}{\underset{r_{c}}{\int }}r_{2}f_{2}dA_{2}=\overset{r_{c}}{\underset{0}{\int }}r_{1}\frac{F_{T}}{\pi r_{1}^{2}}\cdot 2\pi r_{1}dr_{1}+\overset{r_{m}}{\underset{r_{c}}{\int }}r_{2}\frac{F_{T}}{2\pi r_{c}r_{2}-\pi r_{c}^{2}}\cdot 2\pi r_{c}dr_{2}$$

(19) $$M=2F\left(\sin\theta \right)\left(\overset{r_{c}}{\underset{0}{\int }}dr_{1}+\overset{r_{m}}{\underset{r_{c}}{\int }}\frac{r_{2}}{2r_{2}-r_{c}}dr_{2}\right)$$

### 2.2. Design of Experiments

To analyze the impact of depth, angle, width and location of the helical slots on the vibration characteristics of novel LTC ultrasonic horns, the full factorial design of experiment (DOE) was used as presented in Table 1. This DOE involved varying one parameter of interest at a time while maintaining the other parameters as a constant. At first, the effect of the slots’ position on LTC vibration characteristics, such as longitudinal amplification, torsional amplification, Eigenfrequencies, stresses and amplitude ratio, were analyzed for novel LTC horns. Various stress components were normalized pertaining to the material’s yield strength. Subsequently, the effects of depth, angle and width of the slots on the vibration characteristics of novel LTC horns were examined to determine the optimal performance factors in terms of VM stresses and amplitude ratio. Four-factor, four-level orthogonal experiments ($L_{16}$) were developed using the Taguchi technique to examine the synchronized impact of various geometric parameters for improved performance characteristics of ultrasonic LTC horns, as presented in Table 2.

### 2.3. LTC Ultrasonic Horn Designs for Giant Magnetostrictive Transducer

**Figure 4:**
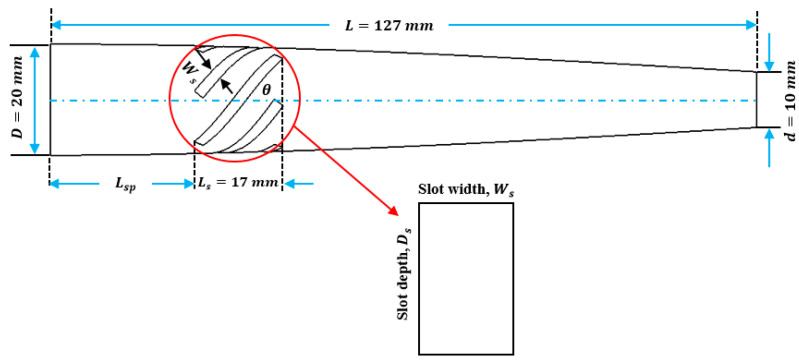
Two-dimensional sketch of novel LTC ultrasonic horn.

For this study, SolidWorks^®^ 2024 was utilized to develop 3D CAD models of novel ultrasonic LTC horns, and ANSYS^®^ 2024 R1 was used for harmonic and modal analyses to numerically compute the LTC vibration characteristics. The optimal LTC horn was selected in terms of amplitude ratio and VM stress. The length, slot length, transducer side diameter and tool end diameter of novel ultrasonic LTC horns were taken as L=127 mm, $L_{sp}=17\;\mathrm{mm}$, D=20 mm and d=10 mm, respectively, as shown in Figure 4, as per the recommendations for ultrasonic tools by Pang et al. [25] and Munir et al. [27] (Table 3).

### 2.4. Finite Element Analysis

The performance and vibration characteristics of novel LTC horns integrated into giant magnetostrictive transducers were evaluated using FEA [15,18,19,28,29,30,31]. Giant magnetostrictive material was modelled keeping in view its analogy to piezoelectric material [18,19]. Equation (20) is used to represent the magneto-electric coupling of the transducer, where ε is strain, $C_{H}$ is elastic coefficient under constant magnetic field strength, β is magnetostrictive strain constant, H is magnetic field strength, *B* is magnetic flux density and μ is magnetic permeability. For magneto-electric coupling problem, (21) is used.

(20) $$\{\begin{array}{c} \sigma =C_{H}\varepsilon -\beta H\\ B=\beta \varepsilon +\mu H \end{array}$$

(21) $$\left[\begin{array}{cc} \left[M\right] & \left[0\right]\\ \left[0\right] & \left[0\right] \end{array}\right]\left[\begin{array}{c} \left[\ddot{u}\right]\\ \left[\ddot{A_{t}}\right] \end{array}\right]+\left[\begin{array}{cc} \left[C\right] & \left[0\right]\\ \left[0\right] & \left[0\right] \end{array}\right]\left[\begin{array}{c} \left[\dot{u}\right]\\ \left[\dot{A_{t}}\right] \end{array}\right]+\left[\begin{array}{cc} \left[K\right] & \left[K_{m}\right]\\ {\left[K_{m}\right]}^{T} & \left[K_{\mu }\right] \end{array}\right]\left[\begin{array}{c} \left[u\right]\\ \left[A_{t}\right] \end{array}\right]=\left[\begin{array}{c} \left[F\right]\\ \left[\Phi \right] \end{array}\right]$$

where [M], [K], [*C*], [F] and [u] represent the mass, stiffness, damping force and displacement matrices, respectively, $\left[A_{t}\right]$ indicates the number of excitation ampere turns on giant magnetostrictive material, [Φ] is the magnetic flux matrix and $\left[K_{m}\right]$ and $\left[K_{\mu }\right]$ are the magnetostrictive coupling and permeability matrices, respectively.

**Figure 5:**
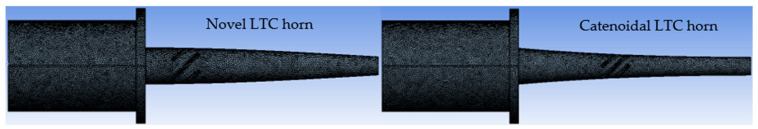
Meshed models of novel ultrasonic LTC horns.

The 3D geometric models were meshed using higher-order tetrahedral elements with a size of 1 mm, resulting in 68,678 elements and 116,235 nodes as per mesh independence analysis due to the ability to deliver better results in comparison to their linear counterparts [15,18,19,28,29,30,31], as illustrated in Figure 5. To analyze the harmonic excitation response (HER) of novel LTC ultrasonic horns for giant magnetostrictive transducers using FEA, harmonic and modal analyses were executed within the 10–30 kHz frequency range. In modal analysis, emphasis was placed on the LTC modes and the corresponding Eigen-frequencies, due to their usefulness in enhancing the surface quality of brittle materials [22,25,32], using a block Lanczos Eigensolver. The torsional ($T_{A}$) and longitudinal ($L_{A}$) vibration amplitudes, stresses and amplitude ratio ($T_{A}/L_{A}$) in novel LTC ultrasonic horns were computed using the full-solution method of harmonic analysis [15,27].

### 2.5. Boundary Conditions and Material Properties for Novel LTC Horns

Aluminum (Al), steel (St) and titanium (Ti) are typically employed for novel LTC ultrasonic horns, whereas Terfenol-D is used for giant magnetostrictive transducers, with their respective properties presented in Table 4. The novel LTC ultrasonic horns were provided 20 kHz harmonic excitation with 15 μm longitudinal vibration amplitude by the ultrasonic transducer, without any tangential and radial excitations.

(22) $$v_{x}=15\;\mu m,\;\;\;\;\;v_{r}=v_{y}=v_{z}=0$$

### 2.6. Validation

**Figure 6:**
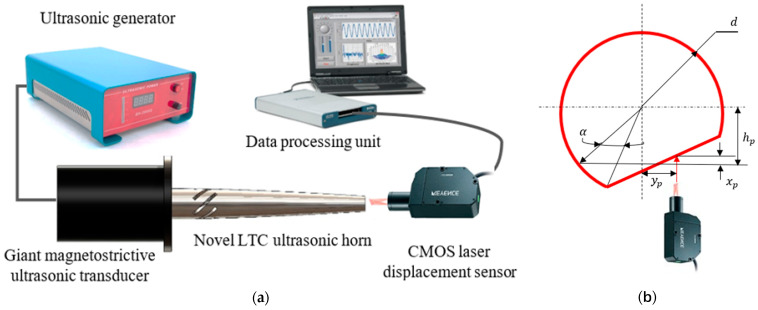
Typical measurement methods for (**a**) longitudinal and (**b**) torsional amplitudes.

The numerical results of this study were validated by comparing them with the experimental outcomes of the optimal LTC stepped horn proposed by Pang et al. [25]. To measure the longitudinal vibration amplitude, a typical experimental setup is used comprising a laser displacement sensor with a data processing unit (Figure 6a). To measure the torsional vibration amplitude, a side plane is milled at the tool end of an LTC horn following the testing technique shown in Figure 6b. The vibration amplitude of the side plane ($A_{s}$) satisfied the following equation:

(23) $$\left[\left\{\left(h_{p}/\cos\alpha \right)-h_{p}\right\}/\tan\alpha \right]+\left(A_{s}/\tan\alpha \right)=y_{p}$$

where $y_{p}$ is the location of measurement point and $h_{p}$ is the height of the side plane. The torsional vibrations are very small; therefore, small angle approximation (tanα≈α and cosα≈1) can be used to simplify the governing equation.

(24) $$\alpha =A_{s}/y_{p}$$

The torsional vibration amplitude can be determined using the following equation:

(25) $$A_{T}=d\alpha /2=dA_{s}/2y_{p}$$

**Figure 7:**
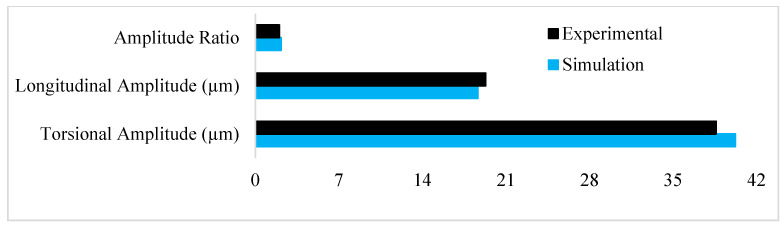
Validation of results [25,27].

To verify the methodology, the optimal LTC stepped horn was remodeled and analyzed using FEA. The FEA outcomes closely matched the experimental results, with errors of only 3.4% and 4.2% in the simulated longitudinal and torsional vibration amplitudes, respectively, as presented in Figure 7. Subsequently, the novel LTC ultrasonic horns for a giant magnetostrictive transducer were analyzed using the experimental conditions reported by Pang et al. [25] for comparison with the optimal LTC stepped horn.

## 3. Results and Discussion

**Figure 8:**
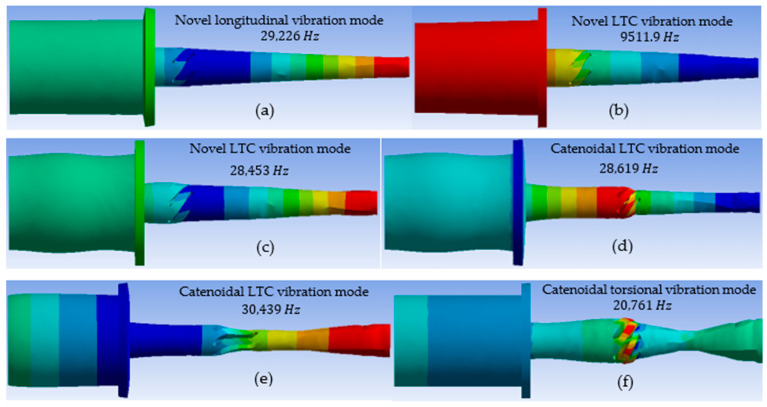
LTC mode extraction of novel ultrasonic tool.

When designing the proposed LTC ultrasonic horns, it is crucial to consider the profile and geometric factors, such as the angle, length, width, depth and position of the helical slots, to attain optimal LTC vibratory performance characteristics. This research shows that a larger amplitude ratio ($T_{A}/L_{A}$) is essential for good-quality ultrasonic machining of advanced brittle materials. In the present work, FEA was employed to investigate the effects of helical slots’ geometric parameters on the HER of the novel LTC ultrasonic horns in terms of Eigenfrequencies, torsional and longitudinal amplitudes of vibrations, stresses and amplitude ratio. The results of the finite element simulations for the mode extractions for the novel LTC ultrasonic horns for a giant magnetostrictive transducer are shown in Figure 8a–f.

**Figure 9:**
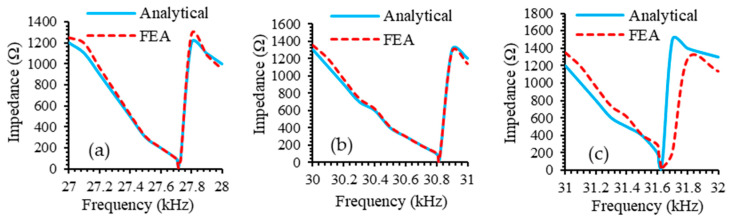
Impedance of LTC ultrasonic tool with (**a**) titanium, (**b**) aluminum and (**c**) steel horns.

The relationship between impedance and frequency for an LTC ultrasonic tool provides valuable insights into its performance and efficiency. As shown in Figure 9, the impedance reaches a minimum value at the Eigenfrequency, where the LTC ultrasonic tool operates most efficiently, with maximum energy transfer from the magnetostrictive transducer to the horn and subsequently to the cutting tool.

### 3.1. Effect of Position of Helical Slots on Novel Ultrasonic LTC Horns

**Figure 10:**
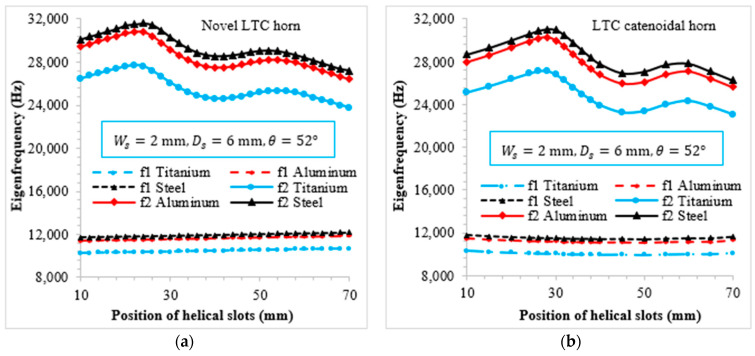
Variation of Eigenfrequencies with helical slot position for various materials: (**a**) novel LTC horn; (**b**) LTC catenoidal horn.

Figure 10 illustrates the relationship of the first and second LTC Eigenfrequencies with the slots’ position for novel LTC ultrasonic horns. The first LTC Eigenfrequency (f1) varies almost linearly with the position of helical slots and falls within the range of 9–12 kHz. These frequencies were found to be well below the excitation ultrasonic frequency (20 kHz) and were therefore excluded from further analysis. Conversely, the second LTC Eigenfrequency (f2) for both horns falls in the range of 24–30 Hz, indicating suitability for ultrasonic applications. Additionally, despite having identical end diameters, lengths and boundary conditions, the Eigenfrequencies for the novel LTC ultrasonic horns differed due to variations in their geometric profiles, which resulted in differences in mass and stiffness.

**Figure 11:**
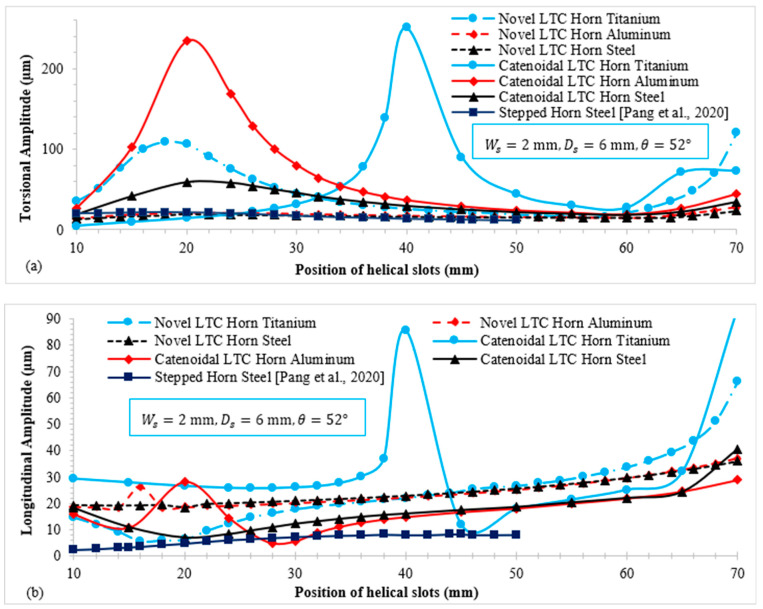
Variation in (**a**) torsional amplitude; and (**b**) longitudinal amplitude with the slot position for various materials [25].

Figure 11 illustrates the relationship of torsional ($T_{A}$) and longitudinal amplitudes ($L_{A}$) with the slot position ($L_{sp}$) for novel LTC ultrasonic horns. For each material, both types of LTC ultrasonic horn profile display similar patterns of amplitude variation, though they differ in magnitude. Figure 11a shows that under identical boundary conditions, catenoidal LTC horns achieve their maximum torsional amplitude sooner than novel LTC ultrasonic horns for all materials. Notably, certain slot positions yield very high $T_{A}$ magnitudes for both LTC horns. This peak occurs because the Eigenfrequency matches the excitation frequency of the ultrasonic transducer, causing resonance. The differences in the moment of inertia I(r,x) along the rotation axis at the same slot position due to dissimilar geometric configurations, as described in Equation (11), and the material properties account for the variation in $T_{A}$ magnitudes despite identical boundary conditions.

Likewise, Figure 11b illustrates the relationship of $L_{A}$ with the slot position for the novel ultrasonic LTC horns for giant magnetostrictive transducers. For both types of LTC horn, the longitudinal vibration amplitude initially decreases and then increases with the slot position. The differing variations in $L_{A}$ for the two ultrasonic LTC horns under identical end diameters and boundary conditions are due to dissimilar cross-sectional areas A(x) at the same axial distance, as shown in Equation (5). Introducing the helical slots further decreases A(x), causing these variations. The $L_{A}$ of the novel LTC horns was observed to be higher than that of the LTC stepped horn proposed by Pang et al. [25] under similar operating conditions. Table 5 presents the maximum $T_{A}$ and $L_{A}$ magnitudes attained by novel LTC ultrasonic horns for various materials.

### 3.2. Effect of Position of Helical Slots on Amplitude Ratio and Stresses

**Figure 12:**
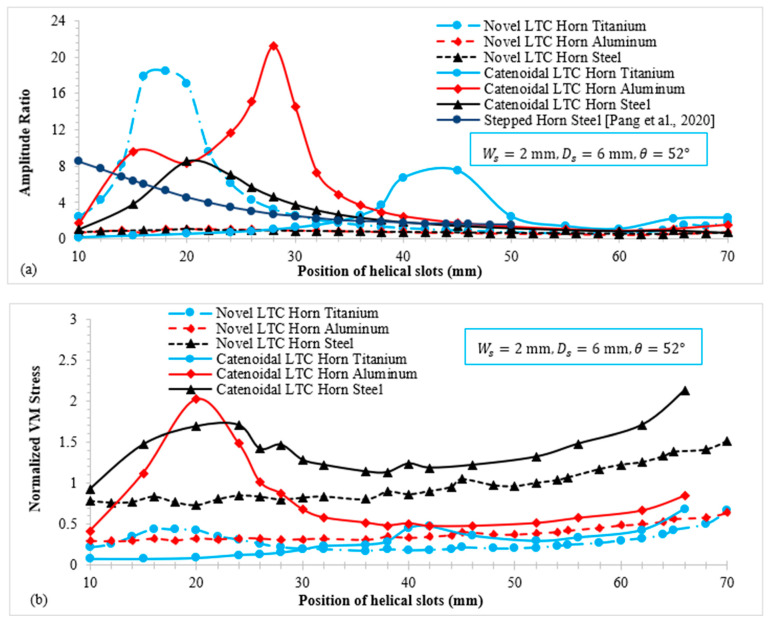
Variation in (**a**) amplitude ratio and (**b**) VM stress with position of helical slots [25].

To compute LTC transformation efficiency of the novel ultrasonic LTC horns, the amplitude ratio ($T_{A}/L_{A}$) was evaluated. Figure 12a,b show the variation in $T_{A}/L_{A}$ and von Mises (VM) stress with the position of the helical slots. In general, the $T_{A}/L_{A}$ for both novel LTC horns initially increases, reaches a maximum and then decreases with the position of the helical slots for all materials. As shown in Figure 9b, VM stress magnitudes in novel LTC ultrasonic horns were observed to rise with the position of the helical slots. Initially, the stresses were found to be less, but they vary as the helical slots move towards the tool end. This is due to the decreasing cross-sectional area of the horn as the slots move towards the smaller end, thereby causing increased stress concentrations.

For the positions of the slots near the tool end, the VM stresses in steel horns were seen to surpass the material’s yield strength ($\sigma_{Y}$). The stresses in aluminum LTC ultrasonic horns were also found to be close to $\sigma_{Y}$ for various geometric parameters. However, the VM stresses in LTC titanium horns remained significantly lower than their respective yield strengths across a wider range of slot positions. Therefore, titanium LTC ultrasonic horns were taken into consideration for further design optimization of the helical slots. In terms of high $T_{A}/L_{A}$ and low stress levels, the optimized position of helical slots for novel and catenoidal LTC ultrasonic horns were found to be 14 mm and 45 mm, respectively.

### 3.3. Effect of Depth of Helical Slots on Novel LTC Ultrasonic Horns

**Figure 13:**
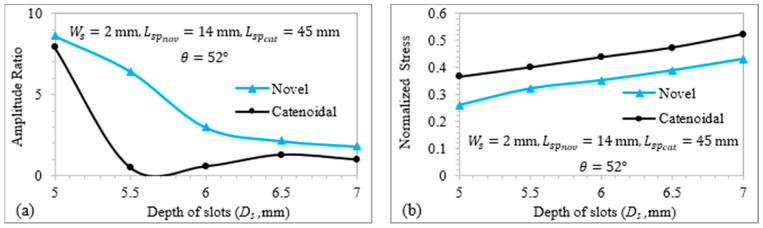
Relationship of (**a**) amplitude ratio and (**b**) normalized VM stress with depth of slots.

Figure 13 illustrates the relationship of $T_{A}/L_{A}$ and VM stress with the slot depth for LTC ultrasonic horns. The optimal depths of slots ($D_{s}$), equal to 44 mm for a novel cubic polynomial ultrasonic LTC horn and 38 mm for a catenoidal LTC horn, were used to achieve a high $T_{A}/L_{A}$ within safe stress levels. As shown in Figure 13a, $T_{A}/L_{A}$ for both LTC ultrasonic horns follows a similar trend: it decreases continuously. Figure 13b shows that VM stress increases with the slot depth for proposed LTC ultrasonic horns, as greater depths result in more material removal from the horn, thereby causing high stress concentrations. The optimal depth of slot to realize high $T_{A}/L_{A}$ with low VM stresses was found to be 5 mm for both LTC ultrasonic horn designs.

### 3.4. Effect of Angle of Helical Slots on Novel Ultrasonic LTC Horns

**Figure 14:**
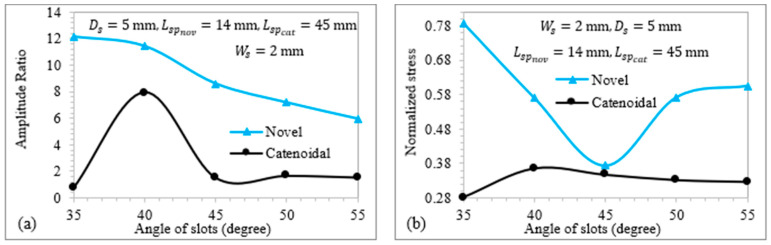
Relationship of (**a**) amplitude ratio and (**b**) normalized VM stress with angle of slots.

Figure 14 shows the relationship of $T_{A}/L_{A}$ and VM stress with the slot angle for the novel LTC ultrasonic horns. The width ($W_{s}$) was set at 2 mm, along with the optimal depths ($D_{s}$) and positions ($L_{sp}$) of the helical slots to ensure a high $T_{A}/L_{A}$ magnitude within safe working stress. Figure 14a indicates that the amplitude ratio generally decreases as the angle of the slots increases. Figure 14b shows that VM stress in the LTC ultrasonic horns decreases with the angle of the helical slots. Increasing the slot angle reduces A(x) at various locations, leading to higher stress levels. The placement of helical slots closer to the transducer side results in smaller A(x) decrement compared to those near the tool end, leading to relatively lower stress concentrations. The optimal angles of helical slots for catenoidal and novel LTC ultrasonic horns were found to be 40° and 45°, respectively, selected for their high amplitude ratios and low VM stresses.

### 3.5. Effect of Width of Helical Slots on Novel LTC Ultrasonic Horns

**Figure 15:**
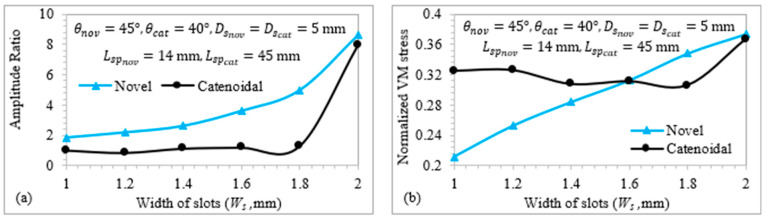
Relationship of (**a**) amplitude ratio and (**b**) normalized VM stress with the slot width.

Figure 15 displays the relationship of $T_{A}/L_{A}$ and VM stress with the slot width for novel LTC horns. As shown in Figure 15a, increasing the slot width causes $T_{A}/L_{A}$ to rise. This is because a wider slot decreases the I(x,r) and A(x) of the LTC ultrasonic horns, which increases both torsional and longitudinal amplitudes according to (8) and (11), thereby raising the amplitude ratio. Figure 15b presents the variation in VM stress with slot width for novel LTC ultrasonic horns. Increasing the slot width reduces A(x) at various locations, leading to higher stress levels. The optimal width of helical slots in novel LTC ultrasonic horns was found to be 2 mm in terms of high amplitude ratios and low VM stresses in accordance with the optimal LTC stepped horn proposed by Pang et al. [25].

### 3.6. Harmonic Excitation Response of Optimal LTC Ultrasonic Horns

**Figure 16:**
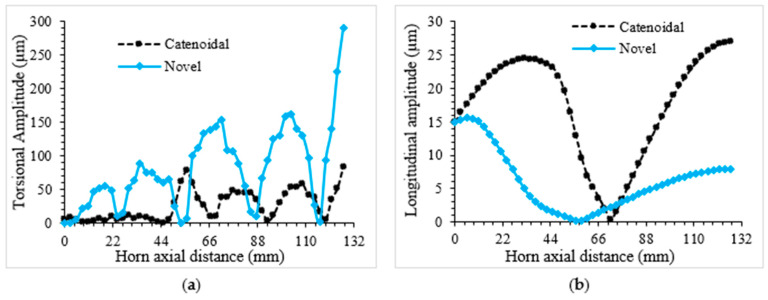
Variation in (**a**) torsional and (**b**) longitudinal amplitudes along optimum LTC horns.

The overall optimal design parameters for novel LTC ultrasonic horns are presented in Table 6. The optimal parameters for a high $T_{A}/L_{A}$ differed for each LTC ultrasonic horn design due to variations in profile, which affect mass, Eigenfrequencies, amplitudes of vibration and amplitude ratio. The deviation of longitudinal and torsional vibration amplitudes for the optimized novel LTC cubic polynomial and catenoidal horns are presented in Figure 16a and Figure 16b, respectively. As depicted in these figures, both vibration amplitudes vary axially, starting from the transducer end and reaching their maximum at the tool end. This performance characteristic is essential for LTC ultrasonic horns to efficiently machine hard and brittle materials.

**Figure 17:**
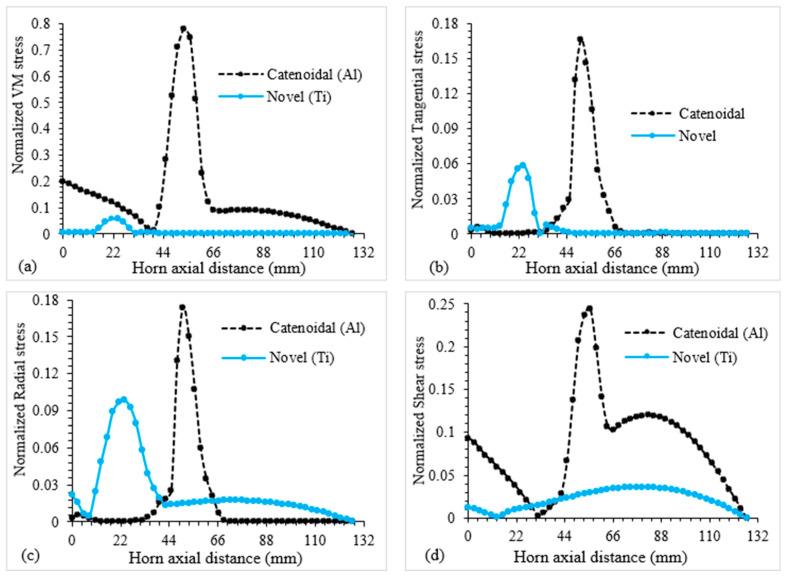
Variation in various stresses along optimal LTC ultrasonic horns.

Figure 17a–d present the deviation in various stresses along with the maximum values, including VM, shear, radial and circumferential stresses, for the two optimal novel LTC ultrasonic horn designs. As depicted in Figure 17a–d, the maximum stress magnitudes occur near the middle of the LTC ultrasonic horns at the slot positions, away from the tool and transducer ends. At input and output ends, the stresses are low and well below the yield strength, ensuring smooth force transmission during robotic RUSM of advanced brittle materials.

### 3.7. Significance Analysis for Novel LTC Ultrasonic Horns

The significance analysis was performed according to the results of $L_{16}$, $4^{4}$ orthogonal experiments as presented in Table 7. The results of the general experiments delivered a range of geometric factors as follows: $L_{sp}=14-50\;\mathrm{mm}$, $D_{s}=5-7\;\mathrm{mm}$, θ=10−25° and $W_{s}=40-55\;\mathrm{mm}$. The variation ranges of $T_{A}/L_{A}$ and normalized VM stresses for the key geometric parameters of the novel LTC horns at various levels were computed to investigate their effect on LTC vibration characteristics (Table 8 and Table 9, respectively). The mathematical representations for variations in terms of position ($R_{L}$), width ($R_{W}$), depth ($R_{D}$) and angle ($R_{\theta }$) of helical slots are written as follows. For amplitude ratio,

(26) $$R_{L_{novel}}\left(15.50\right)>R_{W_{novel}}\left(6.46\right)>R_{D_{novel}}\left(4.65\right)>R_{\theta_{novel}}\left(2.73\right)$$

(27) $$R_{L_{cat}}\left(3.17\right)>R_{\theta_{cat}}\left(1.19\right)>R_{D_{cat}}\left(1.31\right)>R_{W_{cat}}\left(0.93\right)$$

For VM stress,

(28) $$R_{L_{novel}}\left(0.33\right)>R_{W_{novel}}\left(0.22\right)>R_{\theta_{novel}}\left(0.20\right)>R_{D_{novel}}\left(0.18\right)$$

(29) $$R_{L_{cat}}\left(0.37\right)>R_{\theta_{cat}}\left(0.10\right)>R_{W_{cat}}\left(0.13\right)>R_{D_{cat}}\left(0.08\right)$$

For torsional amplitude,

(30) $$R_{L_{novel}}\left(108.88\right)>R_{\theta_{novel}}\left(65.35\right)>R_{D_{novel}}\left(59.69\right)>R_{W_{novel}}\left(50.08\right)$$

(31) $$R_{L_{cat}}\left(87.52\right)>R_{D_{cat}}\left(36.04\right)>R_{\theta_{cat}}\left(34.56\right)>R_{W_{cat}}\left(23.52\right)$$

For longitudinal amplitude,

(32) $$R_{L_{novel}}\left(23.80\right)>R_{W_{novel}}\left(17.34\right)>R_{\theta_{novel}}\left(15.09\right)>R_{D_{novel}}\left(13.71\right)$$

(33) $$R_{L_{cat}}\left(10.53\right)>R_{\theta_{cat}}\left(9.41\right)>R_{W_{cat}}\left(5.21\right)>R_{D_{cat}}\left(4.36\right)$$

**Figure 18:**
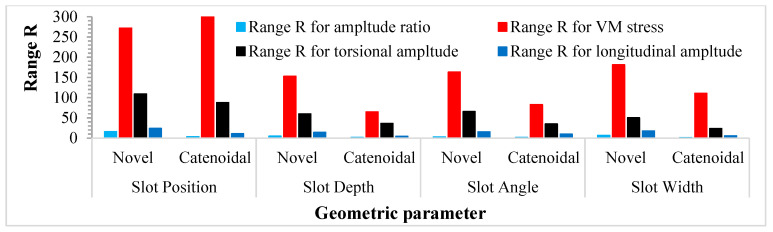
Significant effect of geometric parameters on vibration characteristics of LTC horns.

The evaluation of the range of amplitude ratio variation with various geometric parameters of novel LTC horns revealed the effect of position to be greatest on the amplitude ratio and stresses followed by the width, depth and angle for novel LTC ultrasonic horns, whereas followed by angle, depth and width for catenoidal LTC horns. Similar results were found for torsional and longitudinal amplitudes, as shown in Figure 18.

### 3.8. Performance Assessment of Optimal LTC Horn Designs

For validation purposes, FEA results were compared with experimental outcomes of the optimal LTC stepped horn accessible in the literature. To make sure of the efficacy of FEM used in the present work, the VM and axial stresses corresponding to the optimal $T_{A}/L_{A}$ for the LTC stepped horn [25] were replicated using FEA. The optimal values of $T_{A}$, $L_{A}$ and corresponding stresses for LTC ultrasonic horns for giant magnetostrictive transducers are presented in Table 10.

The LTC stepped horn achieved an optimal $T_{A}/L_{A}$ of 2.0 with $\sigma_{VM}=209.1\;\mathrm{MPa}$. The optimal LTC cubic Bezier horn achieved a $T_{A}/L_{A}$ of 5.18 with $\sigma_{VM}=333.48\;\mathrm{MPa}$. In contrast, the novel LTC ultrasonic horns provided higher amplitude ratios with lower stress concentrations. This high $T_{A}/L_{A}$ combined with low stress concentrations in novel LTC ultrasonic horns will be useful in realizing better-quality machining with lower cutting forces and improved surface morphology for advanced brittle materials, which is essential for efficient robotic RUSM. Various researchers have determined the usefulness of reduced stresses in composite manufacturing as well [33,34].

**Figure 19:**
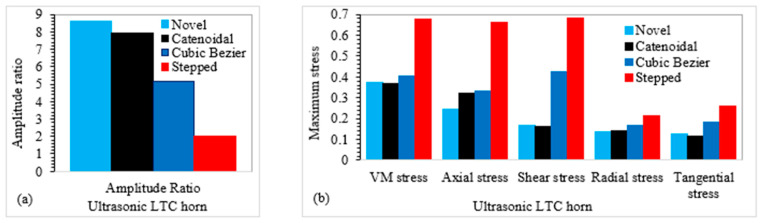
Performance evaluation in terms of (**a**) amplitude ratio and (**b**) stresses for LTC horns.

Figure 19a compares the amplitude ratios of the optimal cubic polynomial and catenoidal LTC horns (8.60 and 7.93, respectively) with the LTC stepped horn, while a comparison of various stresses is presented in Figure 19b. It can be seen that all stress levels in novel LTC horns are lower than those in the similar-sized LTC stepped horn and significantly less than the material’s yield strength, ensuring their usefulness in giant magnetostrictive transducers for robotic RUSM as replacements for the LTC stepped horn.

## 4. Conclusions and Future Aspects

The design optimization of novel cubic polynomial and catenoidal longitudinally-torsionally coupled (LTC) horn designs for giant magnetostrictive transducers was carried out for aluminum, titanium and steel materials by varying the depth, position, angle and width of helical slots. Structural dynamic analysis was performed using ANSYS to numerically compute the Eigenfrequencies and longitudinal and torsional vibration amplitudes as well as various stresses for optimization.

Titanium ultrasonic horns were found to provide good acoustic performance. Generally, the amplitude ratio and torsional amplitude were found to be higher for titanium and aluminum LTC horns compared to those of steel horns. Moreover, the normalized stresses were found to be significantly lower in titanium LTC ultrasonic horns compared to those in aluminum and steel LTC horns.

The significance analysis revealed the effect of the position of the helical slots to be highly significant on the torsional and longitudinal amplitudes, amplitude ratio and VM stresses of LTC ultrasonic horns followed by the width, depth and angle of the helical slots.

The novel cubic polynomial LTC ultrasonic horn is preferable to its catenoidal counterpart, offering a 8.45% higher $T_{A}/L_{A}$. However, the catenoidal LTC ultrasonic horn exhibits 1.87% lower VM stresses. The optimal $T_{A}$ magnitudes for novel LTC cubic polynomial and catenoidal ultrasonic horns were found to be 74.46 µm and 62.48 µm, along with the optimal $L_{A}$ magnitudes of 8.66 µm and 11.67 µm, respectively. The optimal geometric parameters for the novel LTC cubic polynomial ultrasonic horn were $L_{sp}=14\;\mathrm{mm}$, $D_{s}=5\;\mathrm{mm}$, $\theta_{s}=45{}^{\circ}$ and $W_{s}=2\;\mathrm{mm}$, achieving $T_{A}/L_{A}=8.60$. For the novel LTC catenoidal horn, the optimal parameters were found to be $L_{sp}=44\;\mathrm{mm}$, $D_{s}=5\;\mathrm{mm}$, $\theta_{s}=40{}^{\circ}$ and $W_{s}=2\;\mathrm{mm}$, achieving $T_{A}/L_{A}=7.93$. These optimal geometric parameters of helical slots for novel ultrasonic LTC horns resulted in lower stresses compared to the LTC stepped horn.

The results indicate that the novel LTC ultrasonic horns can replace the LTC stepped horn in robotic RUSM for improved vibration characteristics, which would help realize better-quality machining of advanced brittle materials. 

The effect of several factors, such as the number of helical slots around the circumference of the cubic polynomial and catenoidal horns, widths of helical slots greater than 2 mm and depths of helical slots less than 5 mm and greater than 7 mm, were not investigated in this work. It is recommended that future studies explore the effects of these parameters on novel ultrasonic LTC horn designs for giant magnetostrictive transducers. 

## Figures and Tables

**Table 1:** Geometric parameters and conditions for general design of experiments.

Parameter Name	Values	Constant Parameters	
		LTC Catenoidal Horn	Novel LTC Horn
Position of helical slots $\left(L_{sp}\right)$	{10, 12, 14, 16, …, 70 mm}	$W_{s}=2\;\mathrm{mm}$, $D_{s}=6\;\mathrm{mm}$, θ=52°	$W_{s}=2\;\mathrm{mm}$, $D_{s}=6\;\mathrm{mm}$, θ=52°
Depth of helical slots $\left(D_{s}\right)$	{5, 5.5, 6, …, 7 mm}	$W_{s}=2\;\mathrm{mm}$, θ=52°, $L_{sp}=45\;\mathrm{mm}$	$W_{s}=2\;\mathrm{mm}$, θ=52°, $L_{sp}=14\;\mathrm{mm}$
Angle of helical slots (θ)	{35, 40, 45, …, 60°}	$W_{s}=2\;\mathrm{mm}$, $D_{s}=5\;\mathrm{mm}$, $L_{sp}=45\;\mathrm{mm}$	$W_{s}=2\;\mathrm{mm}$, $D_{s}=5\;\mathrm{mm}$, $L_{sp}=14\;\mathrm{mm}$
Width of helical slots $\left(W_{s}\right)$	{1, 1.2, 1.4, …,2 mm}	$D_{s}=5\;\mathrm{mm}$, θ=45°, $L_{sp}=45\;\mathrm{mm}$	$D_{s}=5\;\mathrm{mm}$, θ=45°, $L_{sp}=14\;\mathrm{mm}$

**Table 2:** Geometric parameters and levels for orthogonal experiments.

Level	$\mathrm{Position}\;\mathrm{of}\;\mathrm{Slots}\;(L_{sp})\;\;$ mm	$\mathrm{Depth}\;\mathrm{of}\;\mathrm{Slots}\;(D_{s})$ mm/min	Angle of Slots (θ) degree	$\mathrm{Width}\;\mathrm{of}\;\mathrm{Slots}\;(W_{s})$ mm
1	14	5	40	1.4
2	26	5.5	45	1.6
3	38	6	50	1.8
4	50	6.5	55	2

**Table 3:** Various LTC horn designs with their geometric and mathematical models.

LTC Horn	Geometry	Mathematical Model
Cubic polynomial [15,28] 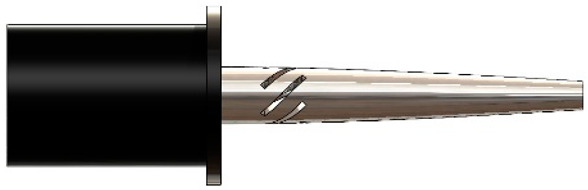	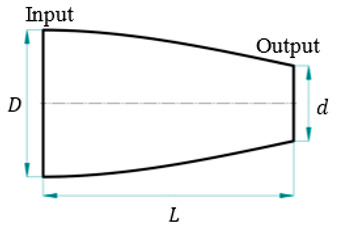	$r=\frac{1}{2}D-\frac{2}{L^{3}}\left(D-d\right)x^{3}+\frac{3}{2L^{4}}\left(D-d\right)x^{4}$
Catenoidal [14,15] 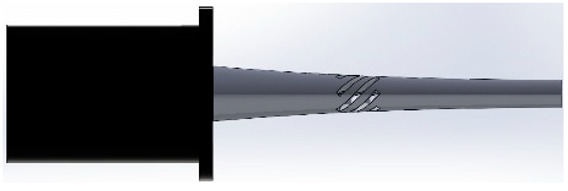	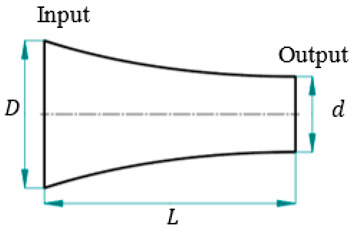	r(x)=dcosha(x−L)
Stepped [15,25] 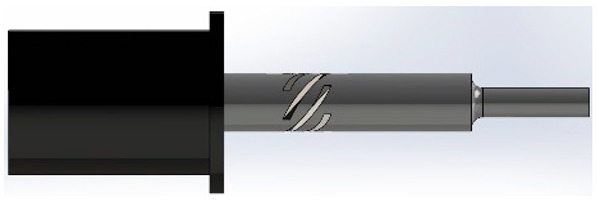	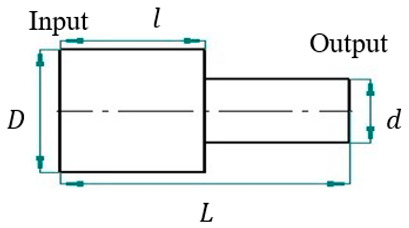	$r\left\{x\in \langle 0;l\rangle \right\}=\frac{D}{2}$ $r\left\{x\in \langle l;L\rangle \right\}=\frac{d}{2}$

**Table 4:** Mechanical properties of giant magnetostrictive transducer and LTC horn materials.

Material	Properties	Value	
Terfenol-D	Piezomagnetism (m/A)	$\left[d\right]=\left[\begin{array}{ccc} 0 & 0 & d_{31}\\ 0 & 0 & d_{31}\\ 0 & 0 & d_{33}\\ 0 & d_{15} & 0\\ d_{15} & 0 & 0\\ 0 & 0 & 0 \end{array}\right]=10^{-9}\times \left[\begin{array}{ccc} 0 & 0 & -5.3\\ 0 & 0 & -5.3\\ 0 & 0 & 11\\ 0 & 28 & 0\\ 28 & 0 & 0\\ 0 & 0 & 0 \end{array}\right]$ $\begin{array}{c} \mathrm{Relates}\;\mathrm{the}\;\mathrm{strain}\;\mathrm{developed}\;\mathrm{in}\;\mathrm{magnetostrictive}\;\\ \mathrm{material}\;\mathrm{to}\;\mathrm{applied}\;\mathrm{magnetic}\;\mathrm{field}. \end{array}$	
	Magnetic permeability (H/m)	$\left[\mu \right]=\left[\begin{array}{ccc} \mu_{11} & 0 & 0\\ 0 & \mu_{22} & 0\\ 0 & 0 & \mu_{33} \end{array}\right]=10^{-6}\times \left[\begin{array}{ccc} 6.29 & 0 & 0\\ 0 & 6.29 & 0\\ 0 & 0 & 6.29 \end{array}\right]$ $\begin{array}{c} \mathrm{Measure}\;\mathrm{of}\;\mathrm{magnetic}\;\mathrm{polarizability}\\ \mathrm{of}\;\mathrm{magnetostrictive}\;\mathrm{material}. \end{array}$	
	Elastic compliance $\left(m^{2}/N\right)$	$\left[s\right]=\left[\begin{array}{cccccc} s_{11} & s_{12} & s_{13} & 0 & 0 & 0\\ s_{21} & s_{22} & s_{23} & 0 & 0 & 0\\ s_{31} & s_{32} & s_{33} & 0 & 0 & 0\\ 0 & 0 & 0 & s_{44} & 0 & 0\\ 0 & 0 & 0 & 0 & s_{55} & 0\\ 0 & 0 & 0 & 0 & 0 & s_{66} \end{array}\right]=10^{-12}\times \left[\begin{array}{cccccc} 17.9 & -5.88 & -5.88 & 0 & 0 & 0\\ -5.88 & 17.9 & -5.88 & 0 & 0 & 0\\ -5.88 & -5.88 & 17.9 & 0 & 0 & 0\\ 0 & 0 & 0 & 26.3 & 0 & 0\\ 0 & 0 & 0 & 0 & 26.3 & 0\\ 0 & 0 & 0 & 0 & 0 & 26.3 \end{array}\right]$	
	Density ρ	$\left(\mathrm{kg}/m^{3}\right)$	9250
	Elastic modulus E	(GPa)	55
	Compressive strength	(MPa)	700
	Poisson’s ratio v		0.3
Titanium	Elastic modulus E	(GPa)	96
	Poisson’s ratio v		0.36
	Density ρ	$\left(\mathrm{kg}/m^{3}\right)$	4620
	Yield strength $\sigma_{Y}$	(MPa)	830
Aluminum	Elastic modulus E	(GPa)	71
	Poisson’s ratio v		0.33
	Density ρ	$\left(\mathrm{kg}/m^{3}\right)$	2770
	Yield strength $\sigma_{Y}$	(MPa)	280
Steel	Elastic modulus E	(GPa)	210
	Poisson’s ratio v		0.27
	Density ρ	$\left(\mathrm{kg}/m^{3}\right)$	7850
	Yield strength $\sigma_{Y}$	(MPa)	310

**Table 5:** Maximum torsional and longitudinal amplitudes for novel LTC ultrasonic horns.

Ultrasonic Horn	$\mathrm{Maximum}\;\mathrm{Torsional}\;\mathrm{Amplitude}\;T_{A}$ (μm)			$\mathrm{Maximum}\;\mathrm{Longitudinal}\;\mathrm{Amplitude}\;L_{A}$ (μm)		
	Ti	Al	St	Ti	Al	St
Novel LTC	74.46	27.78	23.32	08.66	37.01	35.95
LTC catenoidal	62.48	54.80	34.51	11.67	73.20	40.65
LTC stepped [25]	9.6817	198.34	38.60	25.12	19.81	19.30
LTC cubic Bezier [27]	63.460	28.49	25.92	32.58	32.58	32.36

**Table 6:** Optimal parameters for novel and catenoidal LTC ultrasonic horns.

Horn Type	$L_{sp}$ (mm)	$D_{s}$ (mm)	$\theta_{s}$ (degree)	$W_{s}$ (mm)	($T_{A}/L_{A}$)	Normalized VM Stress
Novel LTC	14	5.0	45	2.0	8.60	0.374
LTC catenoidal	45	5.0	40	2.0	7.93	0.367
LTC stepped [25]	20	6.0	52	2.0	2.00	1.076
LTC cubic Bezier [27]	44	5.5	40	2.0	5.18	0.335

**Table 7:** $L_{16}$, $4^{4}$ experimental conditions along with important results for significance analysis.

Experiment	Geometric Parameters of Helical Slots				Amplitude Ratio		Normalized VM Stress	
	Position (mm)	Depth (mm)	Angle (deg)	Width (mm)	Novel	Catenoidal	Novel	Catenoidal
1	14	5	40	1.4	4.879	0.316	0.324	0.082
2	14	5.5	45	1.6	10.052	0.319	0.870	0.079
3	14	6	50	1.8	3.163	0.319	0.785	0.074
4	14	6.5	55	2	1.911	0.316	0.232	0.066
5	26	5	45	1.8	3.979	2.011	0.252	0.118
6	26	5.5	40	2	16.987	0.779	0.406	0.124
7	26	6	55	1.4	21.455	0.933	0.409	0.164
8	26	6.5	50	1.6	22.884	0.917	0.619	0.141
9	38	5	50	2	1.305	6.165	0.217	0.299
10	38	5.5	55	1.8	1.526	1.772	0.225	0.412
11	38	6	40	1.6	1.893	2.909	0.225	0.344
12	38	6.5	45	1.4	2.283	3.105	0.235	0.717
13	50	5	55	1.6	0.627	2.299	0.304	0.429
14	50	5.5	50	1.4	0.813	2.673	0.334	0.341
15	50	6	45	2	0.927	2.907	0.291	0.283
16	50	6.5	40	1.8	0.930	2.703	0.226	0.250

**Table 8:** Comparison of variation in amplitude ratio range for LTC horn designs.

Level	Position of Slots (mm)		Depth of Slots (mm)		Angle of Slots (°)		Width of Slots (mm)	
	Novel	Catenoidal	Novel	Catenoidal	Novel	Catenoidal	Novel	Catenoidal
1	5.00	0.32	2.70	2.70	6.17	1.68	7.36	1.76
2	16.33	1.16	7.34	1.39	4.31	2.09	8.86	1.61
3	1.75	3.49	6.86	1.77	7.04	2.52	2.40	1.70
4	0.82	2.65	7.00	1.76	6.38	1.33	5.28	2.54
Range ($R_{AR}$)	15.50	3.17	4.65	1.31	2.73	1.19	6.46	0.93

**Table 9:** Comparison of variation in VM stress range for LTC horn designs.

Level	Position of Slots (mm)		Depth of Slots (mm)		Angle of Slots (°)		Width of Slots (mm)	
	Novel	Catenoidal	Novel	Catenoidal	Novel	Catenoidal	Novel	Catenoidal
1	0.55	0.08	0.27	0.23	0.30	0.20	0.33	0.33
2	0.42	0.14	0.46	0.24	0.41	0.30	0.50	0.25
3	0.23	0.44	0.43	0.22	0.49	0.21	0.37	0.21
4	0.29	0.33	0.33	0.29	0.29	0.27	0.29	0.19
Range ($R_{VM}$)	0.33	0.37	0.18	0.08	0.20	0.10	0.22	0.13

**Table 10:** Comparison of proposed LTC ultrasonic horns with stepped and Bezier horns.

LTC Horn Type	Diameter Ratio (D/d)	$T_{A}/L_{A}$	Normalized VM Stress	Normalized Axial Stress	Normalized Shear Stress	Normalized Radial Stress	Normalized Tangential Stress
Cubic polynomial	2:1	8.60	0.374	0.245	0.167	0.134	0.127
Catenoidal	2:1	7.93	0.367	0.322	0.163	0.143	0.117
Cubic Bezier [27]	2:1	5.18	0.402	0.330	0.423	0.169	0.181
Stepped [25]	2:1	2.00	0.675	0.661	0.684	0.213	0.261

## Data Availability

Data are contained within the article.
